# Identification of CYFIP2 Arg87Cys Ligands via In Silico and In Vitro Approaches

**DOI:** 10.3390/biomedicines12030479

**Published:** 2024-02-21

**Authors:** Ísis Venturi Biembengut, Emanuella de Castro Andreassa, Tatiana A. C. B. de Souza

**Affiliations:** Laboratory for Structural and Computational Proteomics, Carlos Chagas Institute, Fundação Oswaldo Cruz Paraná (Fiocruz-PR), Curitiba 80320-290, Brazil

**Keywords:** CYFIP2, early infantile epileptic encephalopathies, molecular docking, drug repurposing

## Abstract

The advancement of next-generation sequencing has enabled the identification of specific mutations associated with early infantile epileptic encephalopathies (EIEEs). In EIEE, epileptic spasms and seizures that occur since early childhood lead to impaired neurological development. The CYFIP2 p.Arg87Cys variant was recently related to EIEE. CYFIP2 participates in the Wave Regulatory Complex (WRC), which is related to the regulation of actin dynamics. The variant residue is at the interface between the CYFIP2 protein and WAVE1 protein inside the WRC. Thus, the weakening of this interaction induced by the residue modification, which also causes the flexibilization of the loop 80–110 within the CYFIP2 structure, allows the constant activation of the WCR. This study aimed to identify ligands for CYFIP2 p.Arg87Cys and potential therapy targets using in silico in vitro approaches. Models of different CYFIP2 versions were constructed, and molecular docking analyses were conducted. A total of 3946 ligands from the PDE3 and Drugbank databases were screened, leading to the identification of 11 compounds that selectively bind to the variant protein. The impact of binding in CYFIP2 was also evaluated using a thermal stability assay. These findings contribute to a better understanding of CYFIP2’s functional role in pathology and can guide more in vitro experiments, facilitating the development of targeted therapies for CYFIP2-related conditions.

## 1. Introduction

Epidemiological studies have demonstrated the significance of the genetic causes associated with epilepsy [1,2]. De novo mutations have been predominantly found in this type of syndrome, although they often present with considerable heterogeneity, showing specific variations for each patient [3]. Recent advancements in genetic mapping techniques, such as the use of high-throughput sequencing and genome-wide association studies, have greatly facilitated the identification of genetic variants and contributed to a better understanding of the underlying genetic factors implicated in these diseases [4]. However, the development of targeted therapies for these novel mutations remains a challenge, as it necessitates a comprehensive understanding of the biological mechanisms affected by these variants.

In 2018, variants in the Arg87 residue of the CYFIP2 protein were first associated with early-onset epileptic encephalopathy [5]. Epileptic encephalopathies encompass a group of neurological disorders characterized by spasms and disruptions in brain function, resulting in cognitive and neurological impairment in patients. Early-onset epileptic encephalopathy manifests in the first few months of life and affects the neurological development of infants [6]. The human CYFIP2 protein has approximately 145 kDa and shares 95% similarity with its homologous protein, CYFIP1 [7]. Both proteins have been described as interactors of the Fragile X mental retardation protein (FMRP) [8].

CYFIP2 also plays a crucial role in regulating actin dynamics by participating in the Wave Regulatory Complex (WRC) [9]. The WRC comprises proteins WAVE1 (or WAVE2 or WAVE3), CYFIP2 (or CYFIP1), NCKAP1 (or NCKAP1L), ABI1 (or ABI2 or ABI3), and BRK1, playing a role in regulating actin dynamics within the cell [10,11,12,13]. Variants in the Arg87 residue disrupt this regulation, leading to the constant and uncontrolled activation of the WRC [5,14,15]. In our recent in silico molecular dynamic simulations of the CYFIP2 (NM_001037333.3):c.259C>T (p.Arg87Cys) variant, we observed potential flexibilization in the loop 80–110 of the protein. This loop resides at the interface between the CYFIP2 and the WAVE protein within the WRC complex, and its increased flexibility may negatively impact the stability of CYFIP2 and its interactions with the WAVE protein [16]. Additionally, CYFIP2 Arg87 variants may affect its interaction with RNA-binding proteins (RBPs). These variants form clusters within transfected HeLa cells that co-localize with the Argonaute protein, an RBP. Given that CYFIP2 is known to interact with FMRP, another protein involved in translation regulation, the clustering of CYFIP2 could disrupt its function in other contexts beyond the WRC [8,17]. Thus, reversing the structural effects caused by the Arg87 modification in this protein could be crucial for the future development of target-specific therapies.

Pharmacological chaperones represent a class of small molecules that can facilitate proper protein folding. Typically, these chaperones are drugs designed to bind specifically to a particular protein or group of proteins, with the potential to restore correct protein folding in the presence of structural changes caused by mutations, thereby restoring their functionality [18]. As an example, the work of Abramov and colleagues used in silico screening to identify two pharmacological chaperones for Munc18-1 mutants is also associated with neurodevelopmental disorders. They also proved the potential of the identified ligands to reverse the aggregation of Munc18-1 and restore neuronal function [19].

Therefore, the primary aim of this study is to identify potential CYFIP2 p.Arg87Cys ligands. We created models with the different versions of CYFIP2, and molecular docking analyses were performed. In total, 3946 ligands were screened from the following two databases: PDE3 [20] and Drugbank [21]. We identified 11 compounds that potentially bind selectively to the variant protein and could act as pharmacological chaperones, mitigating the effects of structural modification in CYFIP2. The binding of eight of these compounds was also tested in vitro using a thermostability assay.

## 2. Materials and Methods

### 2.1. CYFIP2 Model Preparation

In order to perform molecular docking, CYFIP2 models were constructed since there is no resolved tridimensional structure for CYFIP2. The crystallographic structure of CYFIP1 within the WRC complex (PDB 3P8C) has been previously solved [9]. Given the high sequence identity of 88% between CYFIP1 and CYFIP2 [8], the atomic coordinates of CYFIP1 (PDB 3P8C, chain A) were utilized as a template for the homology modeling of the native CYFIP2 protein and its variants. To achieve this, the HHpred server was employed to perform an alignment based on the reference sequence NP_001032410 for the CYFIP2 WT model and NM_001037333.3 for the CYFIP2 Arg87Cys model, considering the secondary structure of the proteins [22].

The modeling process was carried out using MODELLER software version 9.23 [23]. For each protein version, namely CYFIP2 WT and CYFIP2 Arg87Cys, a total of 50 models were generated. The selection of final models was performed using the MODELLER function that calculates the “DOPE score” (Discrete Optimized Protein Energy). Additionally, the structures were carefully assessed for clashes between atoms with distances smaller than 1.5 Å using the “find clashes/contacts” tool in Chimera software version 1.14 [24]. Furthermore, the geometry of the models was evaluated using the Molprobity tool version 4.5, specifically through the Ramachandran plot [25].

### 2.2. Grid Generation

The position and shape of the box were previously defined to cover the Arg87 region of the protein. Additionally, the COACH algorithm [26] was used for an analysis to predict the possible binding sites for small ligands in the models. This analysis also assisted in defining the box. In Autodock Vina [27], the grid box was set to cover the residues identified using COACH within the region of residue 87 with the following dimensions in Å: center (x, y, z) = (0.48, 39.17, 231.72), dimensions (x, y, z) = (20.77, 19.34, 19.84).

### 2.3. Ligand Preparation

For the ligand selection, a list of 174 drugs from the PDE3 (Prescribable Drugs with Efficacy in Experimental Epilepsies) database was utilized [20]. To broaden the scope of our analysis and ensure a larger diversity of drugs, an additional set of 3772 compounds was randomly selected from the DrugBank database [21]. The decision to opt for a random selection approach, as opposed to a more targeted one, was driven by the absence of the established literature on drugs interacting with CYFIP proteins. Importantly, our aim was to avoid biasing our screening based on certain properties and, instead, capture a broad spectrum of molecules. The ligands were downloaded along with hydrogen atoms incorporated into their structures for a neutral pH.

### 2.4. Virtual Screening

Prior to conducting the molecular docking experiments, the models were prepared for virtual screening. The preparation involved checking for missing atoms and bonds, examining contacts, and performing energy minimization using the “molecular modeling toolkit” (MMTK) package within Chimera software version 1.14 [28]. Energy minimization was performed using the following parameters: force field Amber ff14SB, 100 steepest descent steps, a steepest descent step size of 0.2 Å, 10 conjugate gradient steps, and a conjugate gradient step size of 0.2 Å.

Molecular docking analyses were then conducted with all selected molecules as ligands for both CYFIP2 WT and CYFIP2 Arg87Cys models for the comparison of the results. For this purpose, the “PyRx-virtual screening” tool version 0.8, which includes the AutoDock [29] and AutoDock Vina [27] libraries, was utilized. The “exhaustiveness” parameter was set to 8. For virtual screening, AutoDock Vina considers all ligands as flexible and receptors as rigid.

### 2.5. Analysis

The results of molecular docking were evaluated by comparing the binding affinity scores (kcal/mol), predicted using the scoring function of the AutoDock Vina module, between the CYFIP2 WT and CYFIP2 Arg87Cys models. The ligand selection criteria based on the scores are presented in Table 1. Among the 8 poses tested for each ligand, the one with the highest affinity (the most negative value in kcal/mol) was selected.

The LigPlus software version 2.2.8 [30] was employed for the second assessment of the predicted protein–ligand interactions. This evaluation aimed to compare the interaction points of the ligands with the amino acid residues in both versions of the protein at the position of highest predicted affinity. Furthermore, we examined the possibility of the best-predicted pose for one protein version to fit into the other model. To achieve this, the docking result files were superimposed onto the other model using the “Match-Maker” tool in Chimera software version 1.14 [24]. With this superposition, new models were generated and subjected to the same previous analysis using the LigPlus software version 2.2.8.

### 2.6. Cell Culture

The adapted cellular thermal shift assay was employed via the treatment of SH-SY5Y cells transfected with pEF1-α CYFIP2 WT (sequence NP_001032410) and pEF CYFIP2 p.Arg87Cys (sequence NM_001037333.3) with HA tag. These cells were cultured in 6-well plates with 2 mL of the DMEM medium supplemented with 10% Fetal Bovine Serum (FBS). The cells were grown at 37 °C under a 5% CO_2_ atmosphere incubator. Transfection was carried out using Lipofectamine™ 2000.

Following a 24 h post-transfection period, each well was treated with either 20 μL of DMSO (control groups) or one of the tested drugs (50 μM) for 1 h. Subsequently, the cells were collected in separate tubes, each containing approximately 500,000 cells, and subjected to an adapted CETSA assay [31]. Briefly, the cells were heated for 3 min at different temperatures (36 and 55 °C), and then the heated cells were kept at −80 °C. This process was conducted in duplicates for both transfected cells using CYFIP2 WT or CYFIP2 Arg87Cys plasmids.

Cell lysis was performed via freeze–thaw which was based on the following 3 cycles: cooling in dry ice for 30 s, thawing at 25 °C for 2.5 min, and vortexing for 5 s. To separate the non-denatured and denatured fractions, the samples were centrifuged at 4 °C, 12,000 rcf for 20 min.

### 2.7. Sandwich ELISA Assay

To quantify the non-denatured fraction of CYFIP2 expressed in the samples, we utilized the supernatant obtained for each condition in a Sandwich ELISA assay. Initially, a 96-well plate was sensitized with 1 μg/mL of the anti-CYFIP2 antibody (Abcam, Fremont, CA, USA), diluted in a carbonate–bicarbonate buffer, and incubated at 4 °C for 12 h. Subsequently, the plate was blocked using PBS 1X, 0.05% Tween, and a 5% milk buffer.

Approximately 1100 μg of the protein extract from SH-SY5Y treated cells was added to the sensitized plates and incubated for 1 h at room temperature, with the supernatant of each condition applied to a different well. After washing the wells, the detection was performed using a 1:300 dilution of the detection antibody anti-HA (ThermoFisher Scientific, Waltham, MA, USA), incubated for 1 h at room temperature. The anti-HA antibody specifically aimed to detect the CYFIP2 protein, whether in the WT or Arg87Cys version, overexpressed by the plasmid. Next, the plate was incubated with the pre-diluted (1:10,000) Goat Anti-Mouse IgG (H+L) peroxidase-conjugated antibody (Invitrogen, Waltham, MA, USA) for 1 h at room temperature. Detection involved the use of an O-phenylenediamine dihydrochloride solution, and the absorbance was quantified at an optical density of 490 nm utilizing a Synergy H1 Hybrid Reader (BioTek, Winooski, VT, USA).

Background correction was performed using the average of two blank wells, which was prepared by following the entire process described previously without the addition of the protein extract. Following background correction, the relative change between the drug-treated samples and the control group was calculated. For each sample and each temperature, the DMSO treatment was used as the reference point, with its values subtracted from those for each drug. The duplicates were then averaged, and the standard deviation was determined. This comparative approach facilitated a clearer understanding of the drug-induced effects by providing a relative measure against the baseline DMSO treatment.

Statistical analysis was executed by comparing the absorbance values of each drug (in duplicate) against the absorbance values of the DMSO treatment (also in duplicate) within each sample and temperature condition. To conduct this analysis, we employed a one-way ANOVA function from the scipy.stats library in Python version 2.7.11.

## 3. Results and Discussion

### 3.1. Target Proteins Structure and Properties

In this study, we generated models for both CYFIP2 and CYFIP2 Arg87Cys, aiming to perform a more in-depth structural analysis of this CYFIP2 variant. The final CYFIP2 models were selected based on the lowest DOPE score, indicating better energy optimization of the atom arrangement. Consequently, we obtained structures with a preserved folding pattern similar to that of CYFIP1, with an RMSD of 1.626 Å for CYFIP2 WT and 1.901 Å for CYFIP2 Arg87Cys. Also, if we evaluated only the region within the docking box (residues 78–100, 171–187, 621–639, and 683–691 in the CYFIP1 sequence), we obtained a RMSD of 0.789 Å for CYFIP2 WT and 0.949 Å for CYFIP2 Arg87Cys. The final verification confirmed that the models exhibited no atom clashes and that over 99% of their residues were located in permissible regions according to the Ramachandran plot (Table 2).

**Figure 1:**
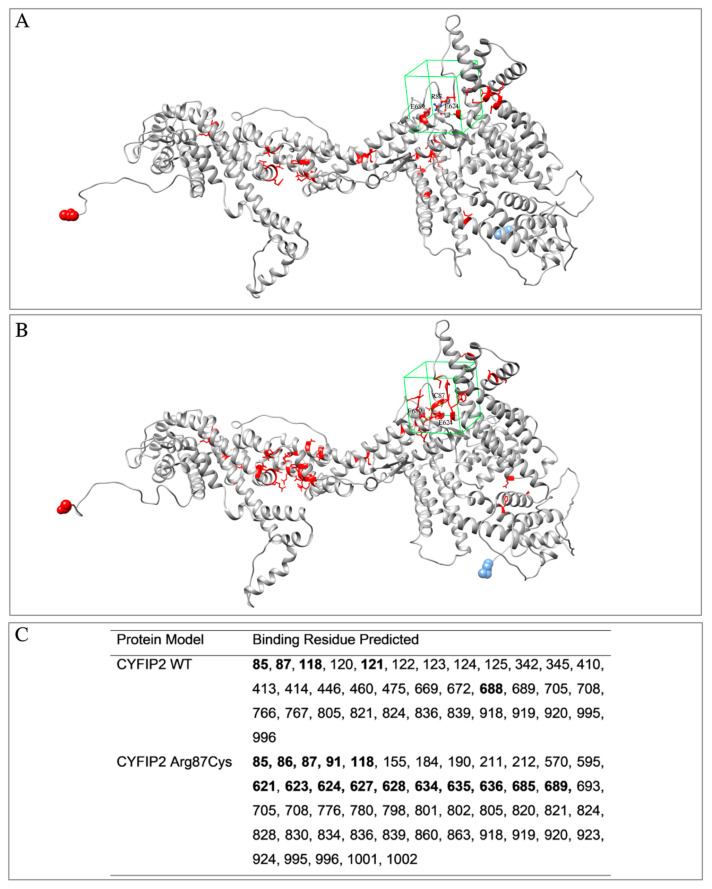
Models of CYFIP2 (in gray) with residues predicted for interaction with ligands using the COACH software (in red). Highlighted is the drawing of the box used in the molecular docking experiments (in green). (**A**) CYFIP2 WT, (**B**) CYFIP2 Arg87Cys. (**C**) Positions of the residues identified as binding sites using the algorithm and residues inside the box for the molecular docking are in boldface. The residue N-terminal and C-terminal are highlighted as blue and red spheres, respectively.

The models were also analyzed using the COACH software (https://zhanggroup.org/COACH/) to investigate potential ligand-binding regions (Figure 1). In this analysis, it was observed that the region encompassing the Arg87 residue of the protein was covered. It was also noted that the predicted binding residues differed in the left lobe regions between the models. As the models were not entirely identical, even in regions not within the mutation, the hybrid sequence and structure comparison method employed for the binding site prediction might be influenced by these subtle structural distinctions. Based on this information, the box for ligand screening in molecular docking was designed to cover residue 87 and other residues determined using the algorithm within the same region in both models.

### 3.2. Ligand Selection and Molecular Docking

Drug repurposing is a strategy used to leverage molecules that have already been studied for one pharmacological purpose, many of which are already used in clinical practice, for a new use [32]. When there is a clear target, molecular docking can be employed for an initial screening of these molecules. This approach reduces the costs associated with the discovery of new compounds and accelerates the development of new therapies [33,34].

Considering these factors, we conducted a virtual screening of 3942 compounds against the models of CYFIP2 WT and CYFIP2 Arg87Cys. An initial analysis of the molecular docking results yielded 65 ligands according to the criteria established in Table 1. The results for all scores of the 3946 evaluated ligands in the experiments for both CYFIP2 and CYFIP2 Arg87Cys can be found in Table S1. The 65 selected ligands, along with their CID codes and selection criteria, are listed in Table S2.

### 3.3. Ligand Selection after Refinement

**Figure 2:**
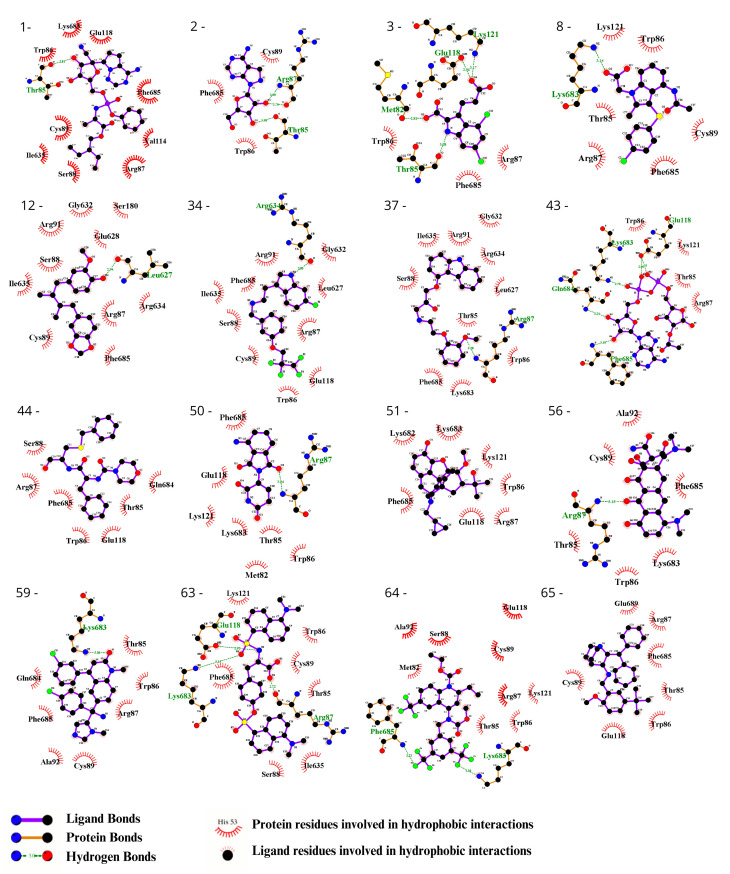
Evaluation using the Ligplot of the 16 ligands selected at the highest affinity position in the molecular docking with the CYFIP2 WT protein. 1—Remdesivir, 2—EXPT02813, 3—Mdl-29951, 8—AZD-1981, 12—Macelignan, 34—Idalopirdine, 37—Carvedilol, 43—EXPT02408, 44—EXPT00813, 50—Pomalidomide, 51—Cyprenorphine, 56—Minocycline, 59—Tipifarnib, 63—N,O-didansyl-l-tyrosine, 64—Torcetrapib, and 65—Maropitant.

**Figure 3:**
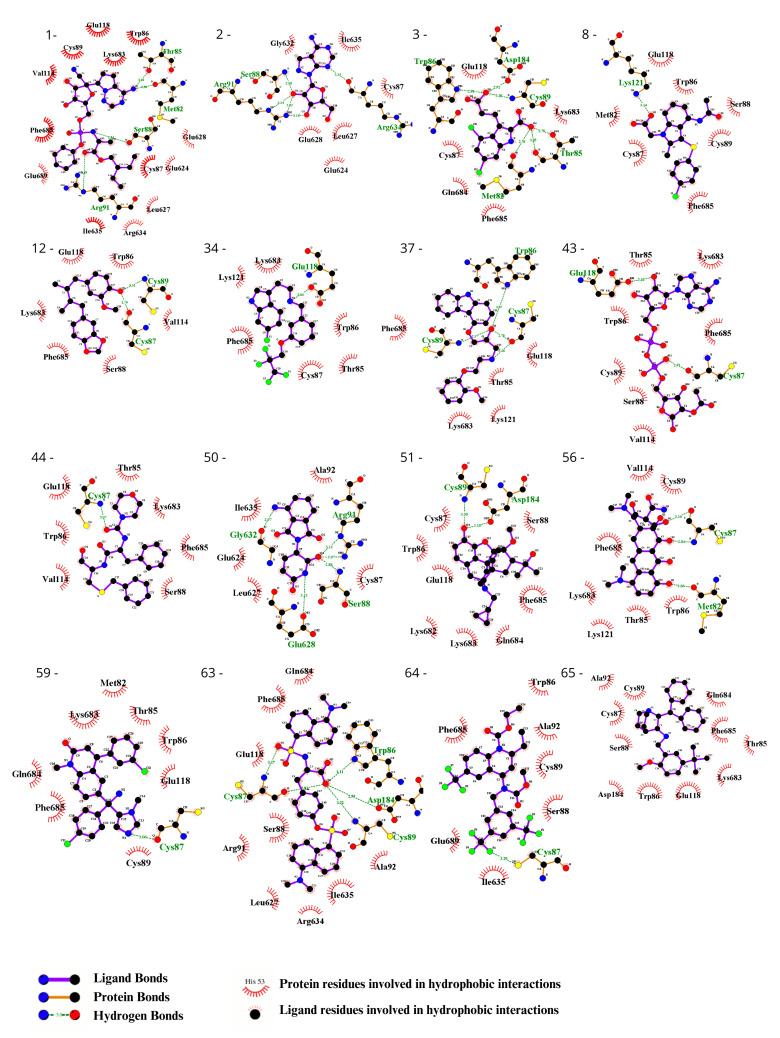
Evaluation using the Ligplot of the 16 ligands selected at the highest affinity position in molecular docking with the CYFIP2 Arg87Cys protein. 1—Remdesivir, 2—EXPT02813, 3—Mdl-29951, 8—AZD-1981, 12—Macelignan, 34—Idalopirdine, 37—Carvedilol, 43—EXPT02408, 44—EXPT00813, 50—Pomalidomide, 51—Cyprenorphine, 56—Minocycline, 59—Tipifarnib, 63—N,O-didansyl-l-tyrosine, 64—Torcetrapib, and 65—Maropitant.

To visually inspect the initial screening, we employed LigPlus software version 2.2.8 to visualize the predicted protein–ligand interactions for the best ligand pose in the molecular docking analysis for the 65 chosen ligands from the first analysis (Figure 2, Figure 3, Figures S1 and S2). In a subsequent analysis aiming to validate the predicted affinity difference between the native and variant protein, we evaluated the fit of the best pose in the native protein when inserted into the variant protein and vice versa (Figures S3 and S4).

**Figure 4:**
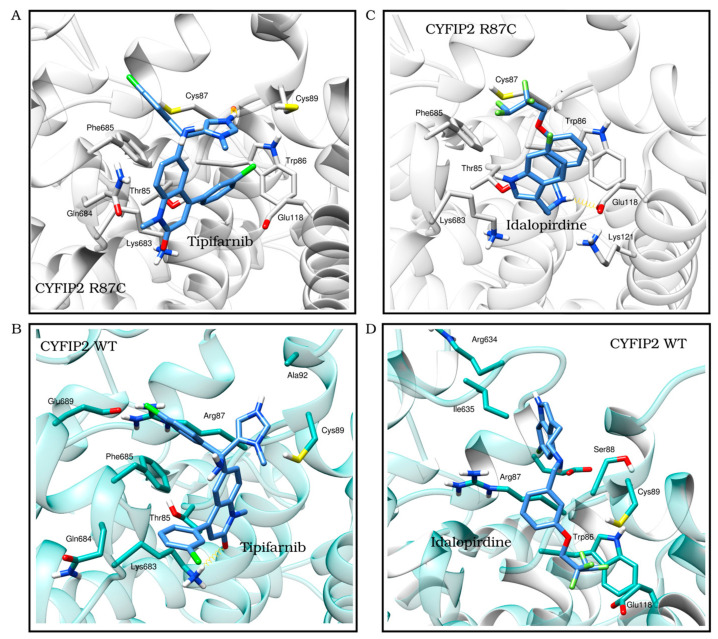
The best docking position of ligands with the best score results from the one selected for CYFIP2 WT and Arg87Cys models. Tipifarnib (binding affinity Arg87Cys: −8.9 kcal/mol, binding affinity WT: −7.6 kcal/mol) in the CYFIP2 Arg87Cys model (**A**) and in the CYFIP2 WT model (**B**). Idalopirdine (binding affinity Arg87Cys: −6.1 kcal/mol, binding affinity WT: −8.3 kcal/mol) in the CYFIP2 Arg87Cys model (**C**) and the CYFIP2 WT model (**D**).

We observed the interactions of the molecules within the structures. By analyzing the molecules in the best position for one of the proteins in the opposite model, we identified interactions that were specific to each model. This allowed us to determine which molecules had screening scores that were more consistent with the observed interactions. Following the refinement of the initial screening, the molecules listed in Table 3 were considered the best potential selective inhibitors for either the variant or native CYFIP2. Figure 4 also shows in detail the interactions resulting from the docking between the models, Tipifarnib (the ligand from Table 3 with the best binding affinity score for CYFIP2 Arg87Cys) and Idalopirdine (the ligand from Table 3 with the best binding affinity score for CYFIP2 WT).

In summary, after this visual inspection of the predicted interactions, a total of 16 compounds were selected, with 11 exhibiting a high predicted affinity for the variant protein. Among these molecules, four (minocycline, pomalidomide, remdesivir, maropitant) are already utilized clinically in other treatments. Given that the variant directly affects the cells of the central nervous system in patients, it is also important to assess whether there is information regarding the passage of these compounds across the blood–brain barrier and whether there is evidence that their long-term use would be safe as a treatment.

Maropitant is an orally administered veterinary medication used to treat nausea in dogs and cats [35] with no evidence of its application in humans. Minocycline is a tetracycline analog antibiotic [36] that has also been identified to have effects on the nervous system and can cross the blood–brain barrier [37]. Clinical studies have been conducted in children for the treatment of autism and Fragile X syndrome using minocycline (Clinical trials: NCT01053156, NCT02680379, NCT04031755). It was approved by the FDA as a medication in 1971, and it is typically administered orally in capsules or topically. In specific cases, intravenous administration may be used. The prolonged use of minocycline is well-tolerated and carries few risks [38]. For in vitro cytotoxicity assays using the myeloid leukemia cell line (HL-60), minocycline exhibited an estimated IC50 of 9.9 μg/mL [39].

Pomalidomide is an immunomodulatory and antineoplastic agent approved for the treatment of certain types of multiple myeloma [40,41]. There is evidence of its ability to cross the blood–brain barrier [42]. As an analog of thalidomide, it is typically administered orally as capsules to adults [43]. Despite its high hematotoxicity in patients, the prolonged use of pomalidomide has shown good tolerability in some clinical cases [44]. Pediatric use has been tested in phase I clinical trials for the treatment of central nervous system tumors with good patient tolerance [45].

Remdesivir is an antiviral medication [46] that was recently approved by the FDA for the treatment of COVID-19 patients [47]. It is typically administered intravenously or via inhalation [48]. In primate tests, it exhibited less than 5% penetration of the blood–brain barrier [46]. Two selected molecules are also undergoing clinical trials as follows: tipifarnib for the treatment of leukemia (Clinical trials: NCT02807272, NCT02210858) and AZD-1981 for the treatment of asthma (Clinical trials: NCT01197794). In summary, the compounds investigated in this study exhibit diverse pharmacological profiles and have been utilized for various medical purposes.

### 3.4. Thermal Stability of CYFIP2

The thermal shift assay is a relevant tool for studying the thermal stabilization of proteins upon ligand binding. This analysis extends to the cellular context, where it is referred to as the cellular thermal shift assay (CETSA). In this technique, cells are treated with a compound of interest, followed by a heating step to denature and precipitate proteins. Subsequently, cell lysis is performed, and the separation of cell debris and aggregates from the soluble protein fraction is carried out [31].

Guided by the docking results, eight compounds were selected (Minocycline, Pomalidomide, Torcetrapib, Tipifarnib, Carvedilol, Macelignan, Mdl-29951, AZD-1981) to evaluate their potential as stabilizers of CYFIP2 using an adapted thermal shift assay. Typically, interactions between ligands and proteins induce changes in protein thermal stability, resulting in modifications to the midpoint denaturation temperature [49]. In our modified thermal shift assay, we expressed CYFIP2 WT and CYFIP2 Arg87Cys proteins, both tagged with an N-terminal HA tag, in SH-SY5Y cells. Treatment with each compound was followed by heating at either 36 °C or 55 °C to compare CYFIP2 denaturation. After separating soluble and insoluble protein fractions for each sample, we employed an ELISA (utilizing both anti-CYFIP2 and anti-HA antibodies) to quantify CYFIP2 in the soluble fraction (Figures S5 and S6).

**Figure 5:**
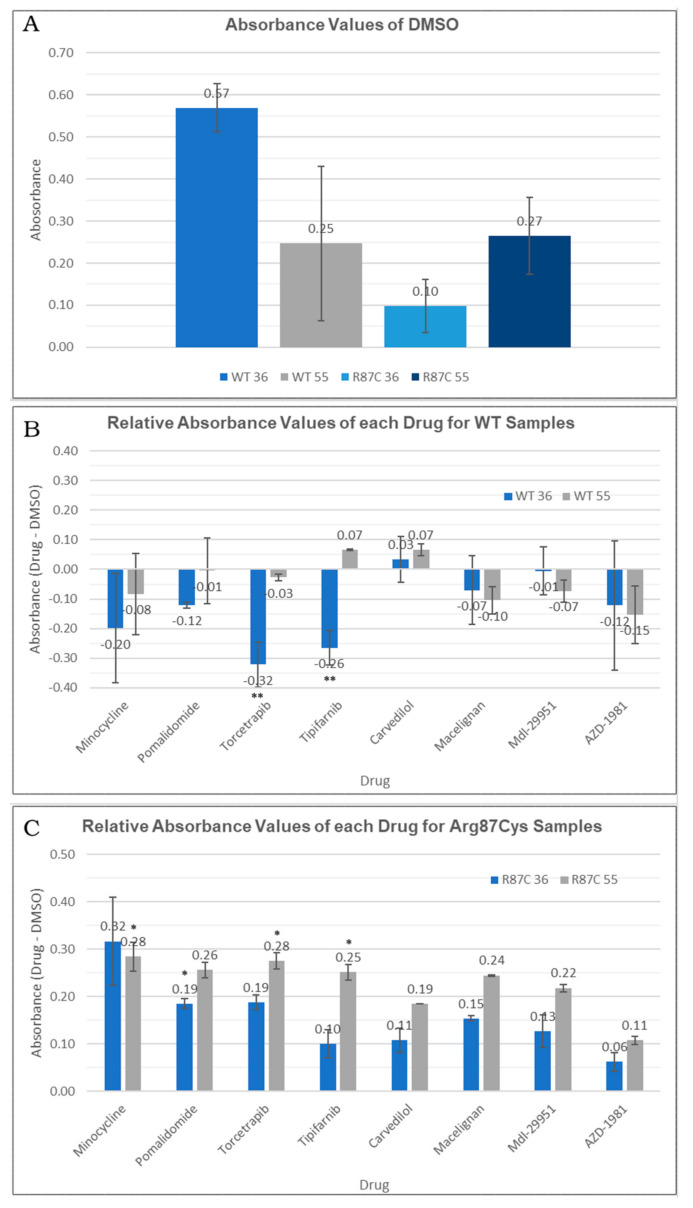
(**A**) Absorbance values for DMSO-treated cells observed in the thermal shift assay for each temperature (36 °C or 55 °C) in each sample (CYFIP2 WT or CYFIP2 Arg87Cys). (**B**,**C**) Relative abundance values (comparing each drug group against the DMSO control group) observed in the thermal shift assay for each temperature (36 °C or 55 °C) in each sample (CYFIP2 WT-B—or CYFIP2 Arg87Cys -C). ** *p*-value < 0.05 * *p*-value < 0.1.

First, we compared the quantification of the soluble fraction between the WT and Arg87Cys groups. This analysis revealed the enhanced stability of CYFIP2 WT compared to CYFIP2 R87C at body temperature (Figure 5A). At 55 °C, both proteins exhibited similar stability behavior (Figure 5A). However, due to our experimental design, when normalizing total protein quantities, direct comparisons of abundances between temperatures are limited. As a result, our analyses are confined within each temperature group or when normalized using the DMSO control.

Next, we conducted a comparison between the non-treated group (where only DMSO was added as a carrier) and each compound tested within the WT and Arg87Cys groups. For the CYFIP2 WT group, none of the compounds increased the protein in the soluble fraction at both tested temperatures. In fact, a significant reduction in CYFIP2 (*p*-value < 0.05) in the soluble fraction was observed at 36 °C upon treatment with torcetrapib and tipifarnib (Figure 5B). By contrast, in the CYFIP2 Arg87Cys group, protein abundance increased in the presence of all ligands at both temperatures (Figure 5C). Specifically, the compounds tipifarnib, torcetrapib, and minocycline showed a significant increase in CYFIP2 Arg87Cys’s abundance at 55 °C, while pomalidomide also showed this at 36 °C (*p*-value < 0.1). Additionally, it is important to note a couple of limitations in our experiments. The abundance of the soluble protein at 36 °C yielded significantly less of the denatured protein compared to 55 °C, given the proximity of 36 °C to body temperature. Consequently, discerning the effects of the compounds at 36 °C with statistical significance becomes challenging, though observed tendencies remain consistent across both temperatures for the Arg87Cys group. In the WT group, certain compounds exhibit varied tendencies at 36 °C and 55 °C; however, higher error bars in this group may potentially mask some results.

Together, these results suggest that certain compounds may interact specifically with the mutated version of the protein (minocycline and pomalidomide), while others (tipifarnib and torcetrapib) can interact in different ways with the wild-type and mutated proteins: they act by enhancing the protein in the soluble fraction-thus promoting stability-or enhancing its denaturation. The distinct interaction modes of these compounds may be related to the predicted site of the interaction. Our prior work [11] showed, through simulations, the flexibilization of the loop comprising residues 80–110 due to the loss of contacts between internal residues in Arg87Cys CYFIP2. Additionally, the key role of residues Arg/Cys87, Glu624, and Glu689 in structural modification was identified. These regions are crucial for restoring the conformation seen in wild-type CYFIP2. Interestingly, our docking analysis identified that tipifarnib might directly bind to five residues of this region, including Cys87, which is positioned near the key residue Glu689.

It is crucial to highlight that the effects of these compounds, selected based on their interaction with CYFIP2 and its variant Arg87Cys (considering patients are heterozygous), necessitate further investigation to determine their potential benefits. Despite their varied applications, our results contribute to target discovery, guiding future in vitro experiments and cell line studies of the pathology model.

## 4. Conclusions

In this study, we developed computational models to investigate CYFIP2 using a homology modeling technique with CYFIP1. These models enabled molecular docking experiments using the following two drug databases: PDE3—174 ligands [20]—and Drugbank—3772 ligands [21]. Through these in silico assays, we identified 11 compounds with the potential for selective interaction with the variant protein, meaning they exhibited a predicted low affinity for CYFIP2 WT and a high affinity for CYFIP2 Arg87Cys. Among them, four compounds are already approved for the treatment of other conditions (Minocycline—an antibiotic—, Remdesivir—an antiviral—, Pomalidomide—an immunomodulator for cancer treatment—, and Maropitant—a receptor blocker for veterinary use against motion sickness).

The identification of compounds that bind to CYFIP2 Arg87Cys may serve as an option to stabilize this structure and attempt to reverse its biological effects.

## Figures and Tables

**Table 1:** The selection criteria for ligands based on the binding affinity score comparison between CYFIP2 WT and CYFIP2 Arg87Cys.

Criteria for Selection of Ligands in the Initial Screening.	
1	Binding affinity score for CYFIP2 WT greater than −6.5 kcal/mol (low affinity); Binding affinity score for CYFIP2 Arg87Cys lower than −6.5 kcal/mol (high affinity); Difference in binding affinity scores between the two proteins greater than 1.0 kcal/mol.
2	Binding affinity score for CYFIP2 WT lower than −6.5 kcal/mol (high affinity); Binding affinity score for CYFIP2 Arg87Cys greater than −6.5 kcal/mol (low affinity); Difference in binding affinity scores between the two proteins greater than 1.0 kcal/mol.
3	Difference in binding affinity scores between the two proteins greater than 1.0 kcal/mol.

**Table 2:** Geometry of CYFIP2 models.

	CYFIP2	CYFIP2 Arg87Cys
Ramachandran outliers	4	5
Ramachandran favored	95.2%	96.3%
Ramachandran allowed	99.7%	99.6%

**Table 3:** List of compounds selected after the refinement of the first screening. The table presents their binding affinities (from the best affinity pose out of the 8 tested) for each of the models, along with their interactions with the model of the protein with the highest affinity.

Ligand	Binding Affinity WT (kcal/mol)	Binding Affinity Arg87Cys (kcal/mol)	Hydrogen Bonds (for the Model with Higher Biding Affinity)	Hydrophobic Interactions (for the Model with Higher Biding Affinity)
Tipifarnib	−7.6	−8.9	Cys87	Met82, Thr85-Cys87, Cys89, Glu118 and Lys683-Phe685
Minocycline	−7.0	−8.1	Cys87 and Met82	Met82, Thr85, Trp86, Cys87, Cys89, Val114, Lys121, Lys683 and Phe685.
N,O-didansyl-L-tyrosine	−7	−8.1	Trp86, Cys87, Cys89 and Asp184	Trp86-Cys89, Arg91-Ala92, Glu118, Asp184, Leu627, Arg634-Ile635 and Gln684-Phe685
Remdesivir	−6	−8.2	Met82, Thr85, Ser88 and Arg91	Met82, Thr85-Cys89, Arg91, Val114, Glu118, Glu624, Leu627-Glu628, Arg634-Ile635, Lys683, Phe685 and Glu689
Pomalidomide	−7	−8.2	Ser88, Arg91, Glu628 and Gly632	Cys87, Ser88, Arg91, Ala92, Glu624, Leu627, Glu628, Gly632 and Ile635
Torcetrapib	−7	−8	Cys87	Trp86-Cys89, Ala92, Ile635, Phe685 and Glu689
Cyprenorphine	−6.9	−8	Cys89 and Asp184	Trp86-Cys89, Glu118, Asp184 and Lys682-Phe685
Maropitant	−6.5	−7.7	-	Thr85-Cys89, Ala92, Glu118, Asp184 and Lys683-Phe685
AZD-1981	−6.4	−7.5	Lys121	Met82, Trp86-Cys89, Glu118, Lys121 and Phe685
EXPT02813	−5.6	−7.3	Ser88, Arg91 and Arg634	Cys87, Ser88, Arg91, Glu624, Leu627, Glu628, Gly632, Arg634 and Ile635
Mdl-29951	−5.7	−7.1	Met82, Thr85, Trp86, Cys89 and Asp184	Met82, Thr85- Cys87, Cys89, Glu118 and Lys683-Phe685
Carvedilol	−7.3	−6.3	Arg87	Thr85-Ser88, Arg91, Leu627, Gly632-Ile635, Lys683 and Phe685
EXPT00813	−7.7	−6.2	Thr85 and Arg87	Thr85-Arg87, Cys89 and Phe685
EXPT02408	−7.8	−6.1	Glu118 and Lys683-Phe685	Thr85-Arg87, Glu118, Lys121 and Lys683-Phe685
Macelignan	−7.5	−6	Leu627	Arg87-Cys89, Arg91, Ser180, Leu627-Glu628, Gly632, Arg634-Ile635 and Phe685
Idalopirdine	−8.3	−6.1	Arg634	Trp86-Cys89, Arg91, Glu118, Leu627, Gly632, Arg634-Ile635 and Phe685

## Data Availability

Data are contained within the article and Supplementary Materials.

## Supplementary Materials

The following supporting information can be downloaded at: https://www.mdpi.com/article/10.3390/biomedicines12030479/s1, Figure S1: Evaluation in Ligplot of the 65 ligands found in the initial screening at the highest affinity position in docking with the CYFIP2 WT protein; Figure S2: Evaluation in Ligplot of the 65 ligands found in the initial screening at the highest affinity position in docking with the CYFIP2 Arg87Cys protein; Figure S3: Evaluation in Ligplot of the 65 ligands found in the initial screening at the highest affinity position in docking with the CYFIP2 WT protein against the CYFIP2 Arg87Cys protein; Figure S4: Evaluation in Ligplot of the 65 ligands found in the initial screening at the highest affinity position in docking with the CYFIP2 Arg87Cys protein against the CYFIP2 WT protein; Figure S5: Average absorbance values measured in our adapted thermal shift assay for each drug group in the CYFIP2 WT samples; Figure S6: Average absorbance values measured in our adapted thermal shift assay for each drug group in the CYFIP2 Arg87Cys samples; Table S1: Docking Scores; Table S2: List of selected compounds after the initial screening.
