# Supplementary material for: Identification of CYFIP2 Arg87Cys Ligands via In Silico and In Vitro Approaches
Source: Biomedicines. 2024 Feb 21;12(3):479. doi: 10.3390/biomedicines12030479 (PMC10967984; doi:10.3390/biomedicines12030479)
Supplement: Supplementary file 1 [file biomedicines-12-00479-s001.zip › biomedicines-28346250-supplemenatry.pdf]

Article

Identification of CYFIP2 Arg87Cys ligands by *in silico and in vitro* approaches

Ísis Venturi Biembengut [1] Emanuella de Castro Andreassa [1] and Tatiana A. C. B. de Souza [1,*]

[1] Laboratory for Structural and Computational Proteomics, Carlos Chagas Institute, Fundação Oswaldo Cruz Paraná (Fiocruz-PR), Curitiba 80320-290, Brazil
* Correspondence: tatiana.brasil@fiocruz.br

1. Supplementary Materials

Table S2. List of selected compounds after the initial screening.

ID	Compound Name	Database	Compound CID (Pubchem ID)	Binding Affinity (kcal/mol) – CYFIP2 WT	Binding Affinity (Kcal/mol) - CYFIP2 Arg87Cys
1	Remdesivir	Drugbank	121304016	-6.0	-8.2
2	EXPT02813	Drugbank	17754151	-5.6	-7.3
3	Mdl-29951	Drugbank	446916	-5.7	-7.1
4	EXPT01499	Drugbank	448471	-5.2	-6.6
5	Branebrutinib	Drugbank	121293929	-6.1	-7.4
6	Des(carbamimidoyl) zanamivir	Drugbank	445533	-5.4	-6.7
7	EXPT02428	Drugbank	448249	-6.3	-7.4
8	AZD-1981	Drugbank	11292191	-6.4	-7.5
9	EXPT02245	Drugbank	5288855	-5.5	-6.6

10	Porfiromycin	Drugbank	13116	-5.6	-6.6
11	EXPT01096	Drugbank	461279	-5.6	-6.6
12	Macelignan	Drugbank	10404245	-7.5	-6.0
13	Bevenopran	Drugbank	10452732	-7.6	-6.0
14	MBX-8025	Drugbank	11236126	-7.4	-5.6
15	DB08322	Drugbank	11669698	-7.9	-5.9
16	DB08013	Drugbank	130188	-8.2	-6.4
17	DB08784	Drugbank	1432578	-7.9	-6.1
18	DB07355	Drugbank	14368760	-6.5	-4.8
19	DB08297	Drugbank	150889	-7.0	-6.0
20	Melperone	Drugbank	15387	-6.8	-5.7
21	(S)-Fluoxetine	PDE3	1548968	-7.4	-6.4
22	Arverapamil	Drugbank	15593907	-7.1	-5.8
23	DB08193	Drugbank	15876	-6.7	-5.7
24	DB08401	Drugbank	16741209	-7.2	-5.8
25	Cobiprostone	Drugbank	16757521	-7.1	-5.9
26	Hydroxyprogesterone caproate	Drugbank	169870	-7.4	-6.4
27	DB07782	Drugbank	1722	-7.4	-6.0
28	Proxibarbal	Drugbank	17336	-6.7	-5.7
29	Dobesilic acid	Drugbank	17507	-6.9	-5.7
30	Bis-Benzamidine	Drugbank	17753851	-7.8	-6.4
31	Etalocib	Drugbank	177941	-8.5	-6.4
32	EXPT01529	Drugbank	188347	-7.4	-6.1
33	Ramelteon	PDE3	208902	-7.0	-6.0
34	Idalopirdine	Drugbank	21071390	-8.3	-6.1
35	Capromorelin	Drugbank	216208	-7.8	-6.4
36	DB07269	Drugbank	23653503	-7.6	-5.7

37	Carvedilol	Drugbank	2585	-7.3	-6.3
38	Diphenidol	Drugbank	3055	-7.8	-6.3
39	Arformoterol	Drugbank	3083544	-7.0	-5.7
40	EXPT00994	Drugbank	445383	-8.5	-6.4
41	EXPT01967	Drugbank	445569	-7.9	-6.0
42	EXPT00747	Drugbank	446783	-7.0	-5.8
43	EXPT02408	Drugbank	447049	-7.8	-6.1
44	EXPT00813	Drugbank	5287870	-7.7	-6.2
45	EXPT01301	Drugbank	65070	-7.4	-6.4
46	EXPT01184	Drugbank	65103	-7.6	-6.3
47	Aminoglutethimide	PDE3	9UQ	-6.9	-5.9
48	Nisoldipine	PDE3	4499	-5.4	-6.4
49	Protirelin	PDE3	638678	-6.7	-8.1
50	Pomalidomide	Drugbank	134780	-7.0	-8.2
51	Cyprenorphine	Drugbank	20054882	-6.9	-8.0
52	N-Acetylglucosamine	Drugbank	1738118	-4.4	-6.3
53	Misoprostol	PDE3	5282381	-6.6	-5.5
54	Epinephrine	PDE3	5816	-6.5	-5.5
55	Dexmedetomidine	PDE3	5311068	-7.0	-5.9
56	Minocycline	PDE3	54675783	-7.0	-8.1
57	Ataciguat	Drugbank	213037	-8.1	-9.2
58	Sulfamazone	Drugbank	187764	-8.2	-9.2
59	Tipifarnib	Drugbank	159324	-7.6	-8.9
60	Experimental	Drugbank	6323200	-7.4	-8.6
61	Alisertib	Drugbank	24771867	-7.5	-8.6
62	BMS-986158	Drugbank	118196485	-7.3	-8.3
63	N,O-DIDANSYL-L-TYROSINE	Drugbank	446468	-7.0	-8.1

64	Torcetrapib	Drugbank	159325	-7.0	-8.0
65	Maropitant	Drugbank	204108	-6.5	-7.7

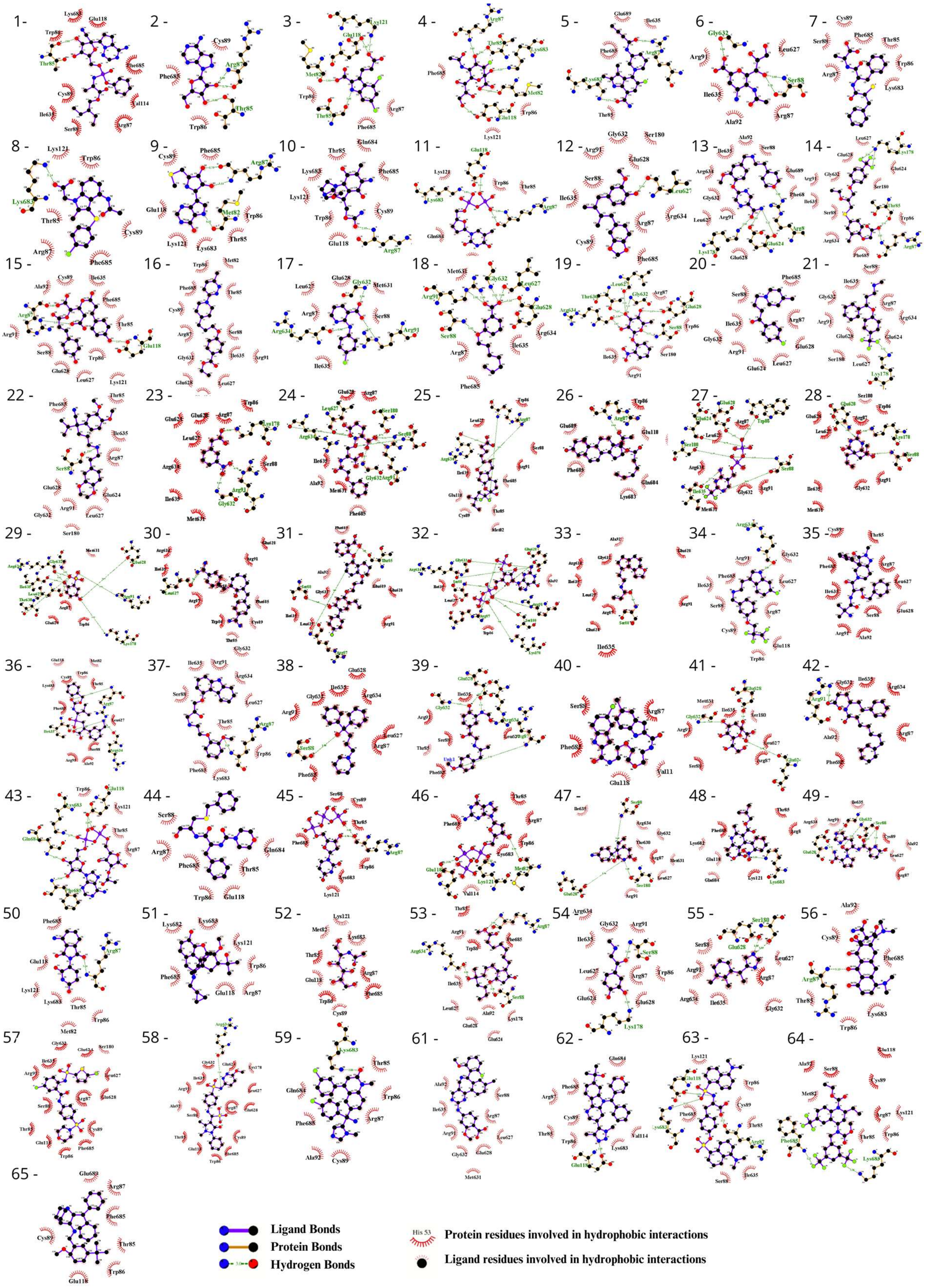

Figure S1. Evaluation in Ligplot of the 65 ligands found in the initial screening at the highest affinity position in docking with the CYFIP2 WT protein. The numbers follow the molecules IDs in table S2.

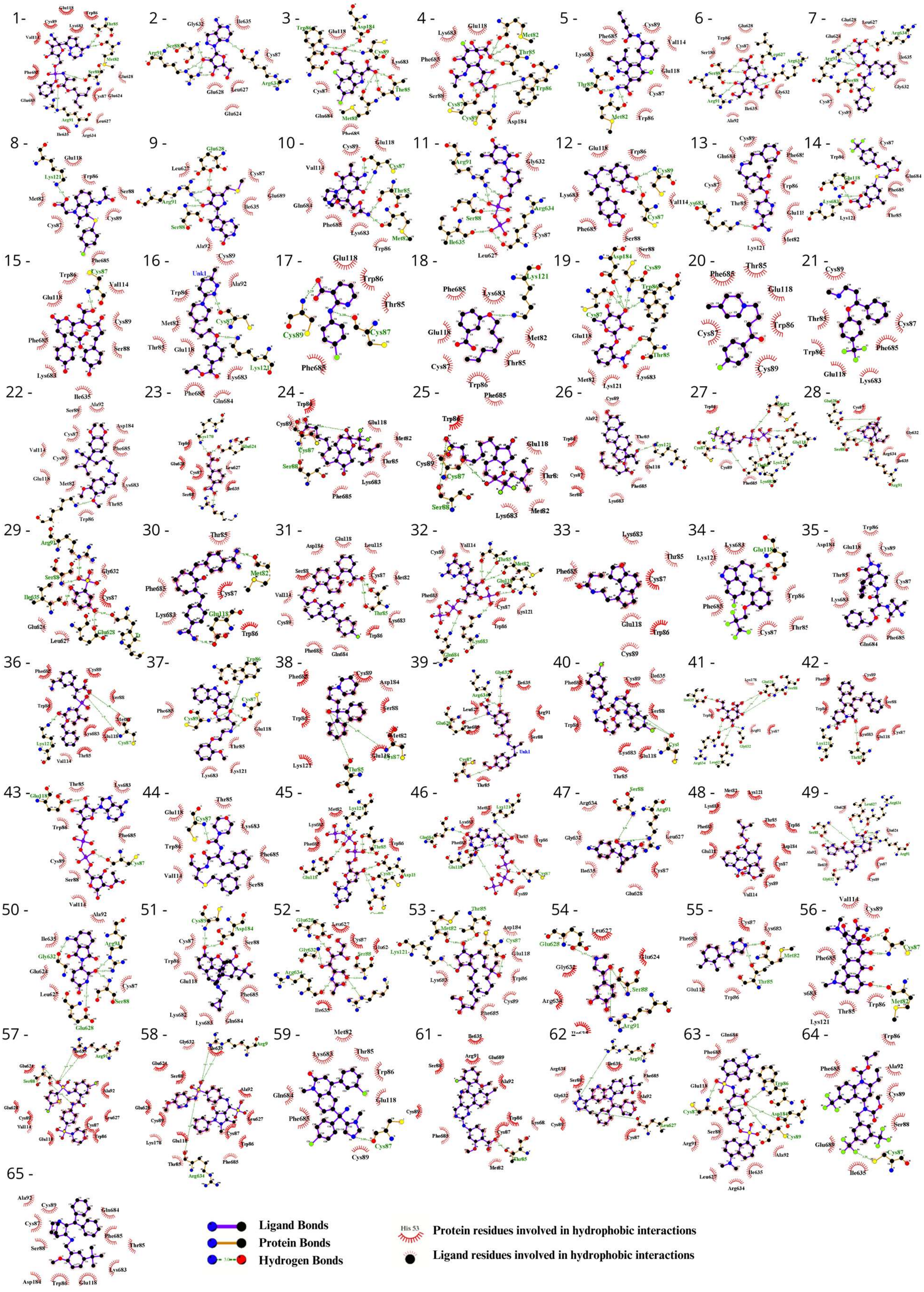

Figure S2. Evaluation in Ligplot of the 65 ligands found in the initial screening at the highest affinity position in docking with the CYFIP2 Arg87Cys protein. The numbers follow the molecules IDs in table S2.

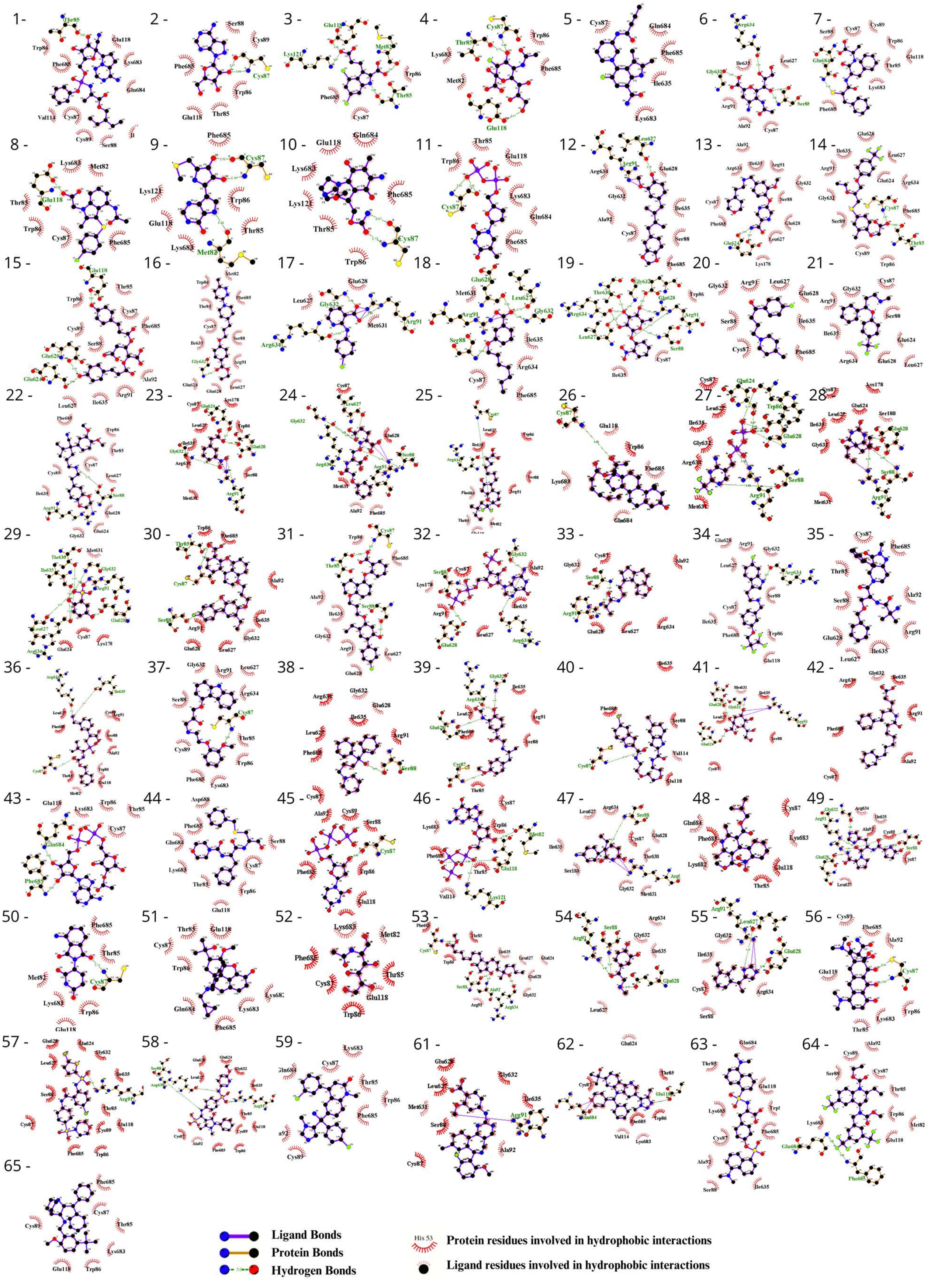

Figure S3. Evaluation in Ligplot of the 65 ligands found in the initial screening at the highest affinity position in docking with the CYFIP2 WT protein against the CYFIP2 Arg87Cys protein. The numbers follow the molecules IDs in table S2.

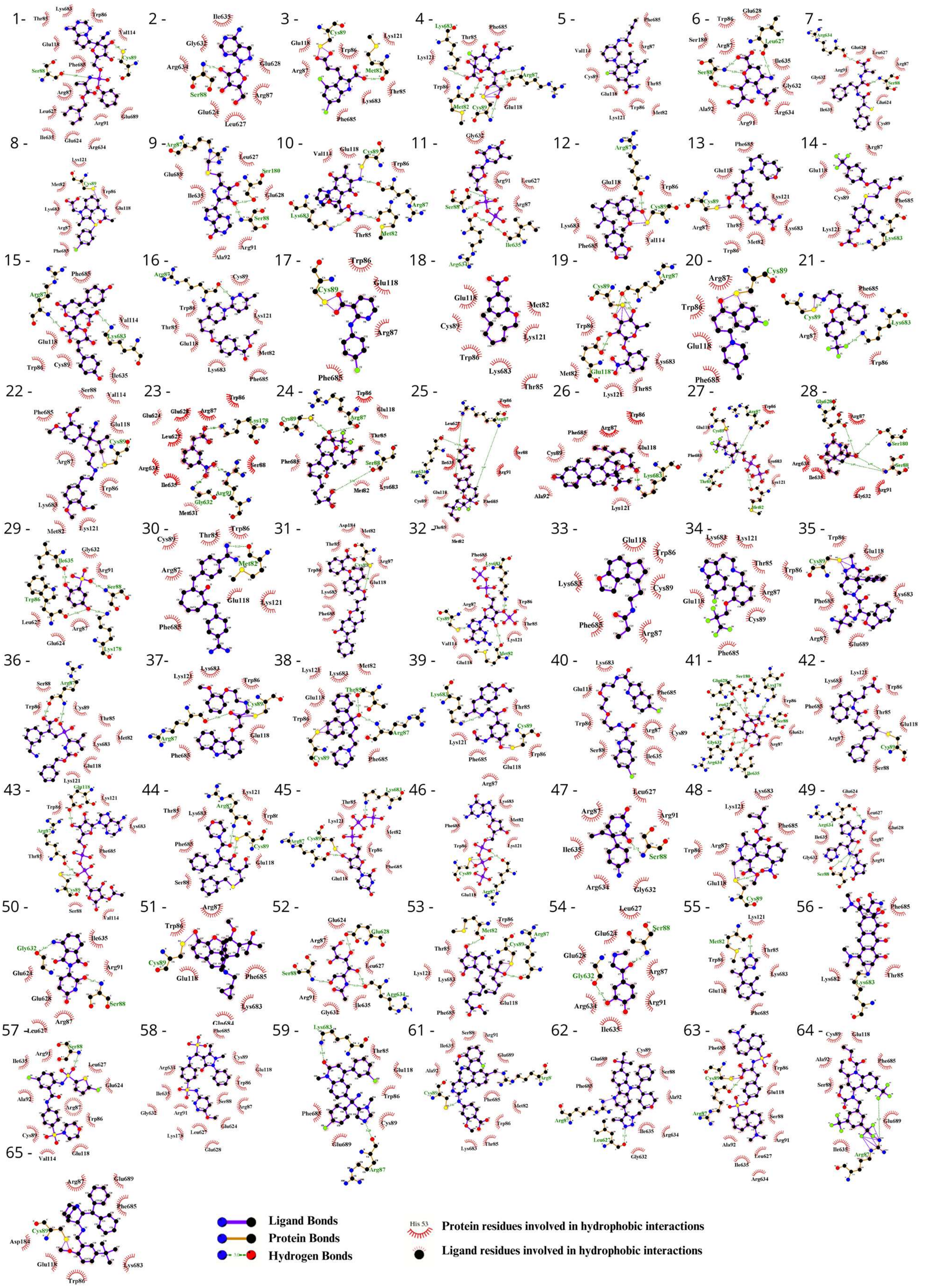

Figure S4. Evaluation in Ligplot of the 65 ligands found in the initial screening at the highest affinity position in docking with the CYFIP2 Arg87Cys protein against the CYFIP2 WT protein. The numbers follow the molecules IDs in table S2.

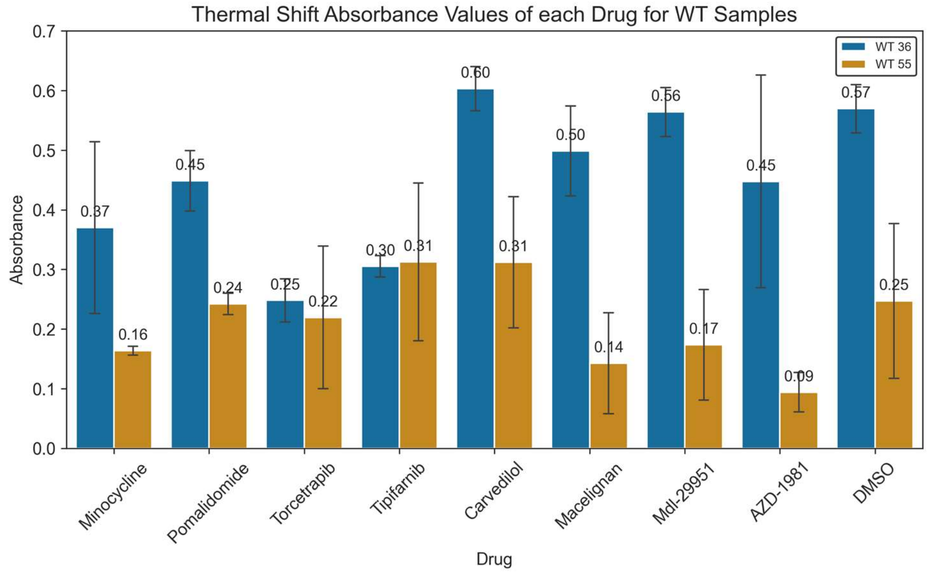

Figure S5. Average absorbance values measured in our adapted thermal shift assay for each drug group in the CYFIP2 WT samples. The error bars represent the standard deviation of the average between the two biological replicates.

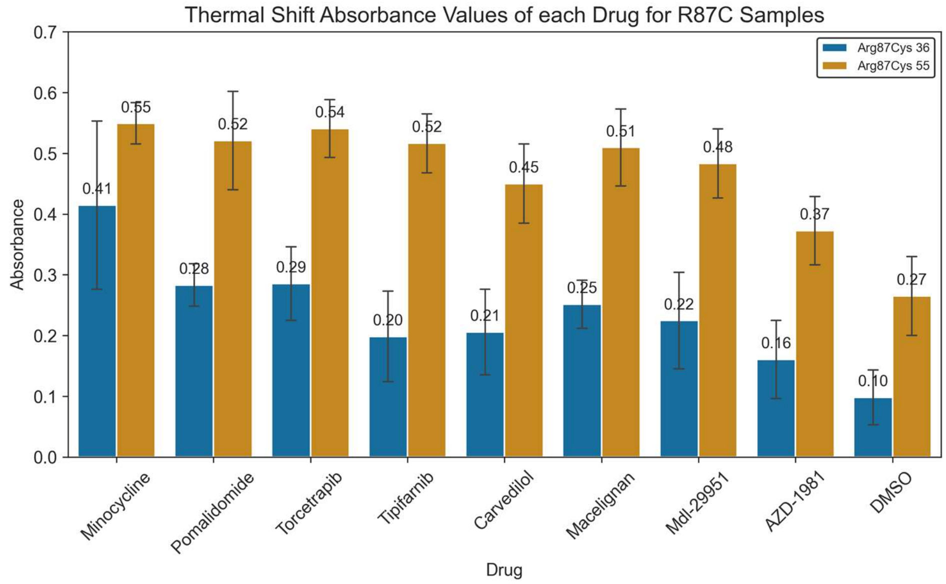

Figure S6. Average absorbance values measured in our adapted thermal shift assay for each drug group in the CYFIP2 Arg87Cys samples. The error bars represent the standard deviation of the average between the two biological replicates.
